# Detection and Characterization of Porcine Sapelovirus in Italian Pig Farms

**DOI:** 10.3390/ani10060966

**Published:** 2020-06-02

**Authors:** Eleonora Chelli, Luca De Sabato, Gabriele Vaccari, Fabio Ostanello, Ilaria Di Bartolo

**Affiliations:** 1 Department of Food Safety, Nutrition and Veterinary Public Health, Istituto Superiore di Sanità, 00161 Rome, Italy; chelli.eleonora@gmail.com (E.C.); gabriele.vaccari@iss.it (G.V.); ilaria.dibartolo@iss.it (I.D.B.); 2 Department of Veterinary Medical Sciences, University of Bologna, Ozzano dell’Emilia, 40126 Bologna, Italy; fabio.ostanello@unibo.it

**Keywords:** porcine sapelovirus, swine, PSV, Italy

## Abstract

**Simple Summary:**

Sapelovirus (PSV) is known to infect pigs asymptomatically but, sporadically, can cause reproductive failure and severe neurologic, enteric, or respiratory signs. Sapelovirus infections have been reported worldwide in pigs. However, information about PSV circulation in Italy is unavailable and rarely investigated across Europe. In this study, we reported the circulation of PSV in three Italian pig farms and added novel information about evolutionary heterogeneity of PSV strains showing a low genetic correlation with the other strains detected worldwide. The present study gives information about PSV circulation in intensive pig farms and highlights the need for further investigation.

**Abstract:**

Porcine sapelovirus (PSV) belongs to the genus *Sapelovirus* of the family Picornaviridae. PSV infects pigs asymptomatically, but it can also cause severe neurologic, enteric, and respiratory symptoms or reproductive failure. Sapelovirus infections have been reported worldwide in pigs. The objective of this study was to investigate the presence and the prevalence of PSV in Italian swine farms in animals of different ages to clarify the occurrence of the infection and the genetic characteristics of circulating strains. In the present study, 92 pools of fecal samples, collected from pigs across three farms, were analyzed by Reverse Transcriptase-polymerase Chain Reaction-PCR (RT-PCR). Fecal pools from young growers (63/64) were found positive for Sapelovirus in all farms while detection in sows (4/28) was observed in only one farm. Phylogenetic analyses of the 19 partial capsid protein nucleotide sequences (*VP1*) (6–7 each farm) enable the classification of the virus sequences into three distinct clades and highlighted the high heterogeneity within one farm. The whole genome sequence obtained from one strain showed the highest correlation with the Italian strain detected in 2015. The study adds novel information about the circulation and heterogeneity of PSV strains in Italy and considering the movement of pigs across Europe would also be informative for other countries.

## 1. Introduction 

Sapelovirus is a non-enveloped virus with a single strand positive-sense RNA genome. The virus within the Picornaviridae family belongs to the genus *Sapelovirus* [1] which contains three species, with a unique genome organization: Sapelovirus A formerly known as porcine sapelovirus (PSV), Sapelovirus B as simian sapelovirus, and Avian sapelovirus represented by duck picornavirus [1]. PSV consists of a single serotype, infects pigs and it is not known to infect humans.

The PSV genome is 7.5–8.3 kb length with the typical picornavirus genome organization, including a single open reading frame (ORF), which encodes for a polyprotein containing 12 mature proteins, structural and functional: a leader protein (L), four structural proteins (VP1–4), and seven nonstructural proteins (2A–C, 3A–D) [2].

PSV is transmitted by the fecal–oral route and has been detected in clinically healthy animals as well as from animals affected by severe symptoms such as diarrhea, pneumonia, reproductive failure, and neurological disorders [3,4,5,6,7].

The virus has been investigated in pigs worldwide with prevalence ranging between 7.1% in India [8] and 71.0% in Hungary [9].

The virus has also been found in wild boars with a prevalence of 6.4% in Spain [10] and 27.8% in the Czech Republic [11]. Co-infection of PSV with other enteric viral pathogens (e.g., Porcine teschovirus, PTV; Porcine Enterovirus, PEV) is frequently reported in both asymptomatic animals or in association with symptoms but information on its role in co-infections is still unavailable [5,8,10,11,12,13,14,15].

Genetic heterogeneity among PSV strains has been reported based on phylogenetic analysis of the *VP1* gene [2,13,16,17], which is a highly heterogeneous region.

To date little information on the occurrence of PSV in Italian pig herds is available. Two studies have been conducted on PSV detection methods, not reporting prevalence but confirming the circulation of PSV among Italian pigs [18,19]. More recently, the first Italian PSV complete genome [20] has been published.

During a study aimed at obtaining hepatitis E virus (HEV) full genomes from pig feces by metagenomics next-generation sequencing (NGS) [21], sequences corresponding to PSV were retrieved in three samples from one farm. Based on this result, we investigated the presence of PSV in Italian pig farms, in animals of different age groups. Overall, 92 pooled fecal samples were analyzed to detect the RNA of PSV by RT-PCR from three farms. Five PSV strains were retrieved from the three farms and typed using the sequences of the partial *VP1* coding region (capsid protein). 

## 2. Materials and Methods

### 2.1. Farms and Samples Collection

In 2012 and 2018, sixty-four pooled fecal samples were collected, from clinical healthy young growers (aged between 1–3 months old) and twenty-eight from sows (animals older than 1 year) of three different farms (A, B, and C) in Northern Italy. Sows from farm C, which was closed down immediately after our study, were not sampled. Neither commercial nor geographical linkages (>100 km apart from each other) exist between the three farms. The fecal samples were collected from three points of each pen floor. Twenty-seven samples (15 from young grower pens and 12 from sows) were collected from farm A (farrow-to-finish herd with 300 sows), thirty-two (16 from young growers and 16 from sows) from farm B (farrow-to weaning herd with 1000 sows), and thirty-three (all young growers) from farm C (parent gilts production herd with 300 sows). Twenty-five to 30 animals were housed in each pen. Sampled pens were located in the same barn for each category; young growers and sows were housed in different buildings. On all farms, grower pigs were managed as a single all-in/all-out (AIAO) cohort with cleaning and disinfection (C&D) between cohort groups. Pools were collected from 40–50% of the pens on farms A and C; 25% of the pens were sampled from farm B.

### 2.2. RNA Extraction

Feces were suspended in double-distilled water (ddH_2_O) (10% w/vol), and clarified by centrifugation at 6000× *g* for 10 min. RNA was extracted from 150 μL of supernatant using a QIAamp Viral RNA Mini Kit (QIAGEN, Milan, Italy) according to the manufacturer’s instructions, eluted in 100 µL of RNase-free water, and stored at −80 °C or used immediately.

### 2.3. PSV-RNA Detection

The RNA was tested for the presence of PSV by reverse-transcription PCR (RT-PCR) amplifying a 270 bp fragment in the conserved 5′ UTR using the primer pair PSV-Fp (5′-CGTGCTCCTTTGGTGATTC-3′) and PSV-Rp (5′-GAAAGAGTAGTAGTAGATTCC-3′) [2].

The RT-PCR was performed using the QIAGEN One-Step RT-PCR Kit (QIAGEN, Milan, Italy), following the manufacturer’s instructions, under the following conditions: retro transcription at 50 °C 30 min, denaturation step at 94 °C for 5 min, followed by 40 cycles at 94 °C for 30 s, 53 °C for 30 s, 72 °C for 15 s, and a final extension at 72 °C for 7 min.

### 2.4. Full Genome Sequencing

Total RNA of three individual fecal samples from farm C, were subjected to the sequence-independent single primer amplification (SISPA) as previously described [21] and the libraries sequenced on an Ion Personal Genome Machine (PGM) in Ion 318 Chip v2 (Thermo Fisher Scientific, Rodano, Italy). The reads obtained by the NGS run were analyzed by Galaxy Aries (Istituto Superiore di Sanità, Rome, Italy) (https://aries.iss.it) as previously described [21].

### 2.5. Nested RT-PCR and Sequencing 

To perform sequencing for phylogenetic analysis, an RT-PCR was designed to amplify the *VP1* gene (PSV capsid protein) which is the genome fragment most used for PSV characterization and most frequently available at the National Centre for Biotechnology Information (NCBI) database (https://www.ncbi.nlm.nih.gov).

Two primer pairs were designed within the *VP1* gene of the PSV by the alignment of 73 PSV complete genomes available online (NCBI) and the PSV complete genome (SwPSV75BO2012) obtained in this study by NGS. The resulting primer pairs were Primer_1_F (5′-ACAGYTAGTGCAGCAGAYAACTT-3′) and Primer_1_R (5′-CTAGTTGGGTGGCAGGGTAA-3′) annealing in the 2202-2224 and 3107-3126 position respectively and a second pair of primer for the nested-PCR: Primer_2_F (5′-AATCCCCACTGACACAGCAT-3′) and Primer_2_R (5′-ACAGYTAGTGCAGCAGAYAACTT-3′) annealing in the 2234-2253 and 3067-3086 position respectively (respect to SwPSV75BO2012). The RT-PCR designed was performed using the One-Step RT-PCR Kit (QIAGEN, Milan, Italy), amplifying 924 bp, under the following conditions: reverse transcription at 50 °C 30 min, denaturation step at 94 °C for 5 min, followed by 40 cycles at 94 °C for 30 s, 58 °C for 30 s, 72 °C for 1 min and 30 s, and a final extension at 72 °C for 7 min. The nested PCR was performed using the GoTaq G2 Flexi DNA Polymerase (Promega, Milan, Italy), amplifying 852 bp, under the following conditions: polymerase activation step at 95 °C for 2 min, followed by 40 cycles at 94 °C for 30 s, 58 °C for 30 s, 72 °C for 1 min and 15 s, and a final extension at 72 °C for 7 min. PCR products were analyzed by 1% agarose gel electrophoresis and visualized under ultraviolet illumination. The amplicons of DNA with the expected size were sequenced by Eurofins Genomics (Eurofins Genomics Germany, Ebersberg, Germany).

### 2.6. Phylogenetic Analysis

The 19 nucleotide sequences obtained were analyzed and edited using the Bionumerics software V.6.5 (Applied Maths, Kortrijk, Belgium) and deposited in GenBank under the accession number: SwPSV3B2018, MN836677; SwPSV14B2018, MN836678; SwPSV2B2018, MN836679; SwPSV1B2018, MN836680; SwPSV4B2018, MN836681; SwPSV13B2018, MN836682; SwPSV71BO2012, MN836665; SwPSV84BO2012, MN836666; SwPSV70BO2012, MN836667; SwPSV73BO2012, MN836668; SwPSV74BO2012, MN836669; SwPSV52BO2012, MN836670; SwPSV56BO2012, MN836671; SwPSV57BO2012, MN836672; SwPSV58BO2012, MN836673; SwPSVS45BO2012, MN836674; SwPSV55BO2012, MN836675; SwPSVS39BO2012, MN836676; SwPSV75BO2012, MN836683. Nucleotide sequence similarity searches were performed using the BLAST server on the NCBI GenBank database [22]. Three maximum likelihood (ML) trees were drawn with the MEGA7 software (www.megasoftwares.com) using the general time-reversible model with a Gamma distribution and invariant sites (GTR+G+I) as suggested by the MEGA7 model test, and 1000 bootstrap replicates. The trees were built using the partial *VP1* sequences (790 nt), full genomes reported on NCBI database including the sole PSV complete genome obtained in this study and the 3CD region (3C^pro^, the cysteine protease and 3D^pol^ the catalytic core of the viral replicase, the RNA-dependent RNA polymerase) [23] obtained from the full genome.

### 2.7. Statistical Analysis

Statistical analysis was performed using SPSS software (SPSS Statistics ver. 25; IBM Corp., Chicago, IL, USA). A comparison of prevalence observed for PSV RNA-positive pooled fecal samples by farm (A, B, and C) and category (young growers and sows), was conducted using the chi-square test. The significant limit was set at *p* < 0.05. Confidence intervals were calculated by the binomial (Clopper–Pearson) “exact” method based on the β distribution.

## 3. Results

To evaluate the PSV circulation in Italian pig farms, after the identification of several reads in an NGS study on HEV in pigs [21], the detection of PSV was conducted on total RNA extracted from a pool of feces collected from three farms by one-step RT-PCR, targeting the conserved 5′UTR. PSV was detected on all the three farms (Table 1).

Comparison of prevalence between the two age classes showed a significantly higher PSV RNA detection (chi-square: 69.7; *p* < 0.001) in pooled samples from young growers (63 out 64; 98.4%; 95% CI: 91.6–99.9) than in sows (4 out of 28; 14.3%; 95% CI: 4.0–32.7).

The pooled fecal samples from young growers were positive in all the farms investigated, with a prevalence of 100% from farm A (15/15; 95% CI: 78.2–100), 100% from farm B (16/16; 95% CI: 79.4–100), and 97% from farm C (32/33; 95% CI: 84.2–99.9). In this category, no significant difference (chi-square: 0.95; *p* = 0.62) in the proportion of PSV positive fecal pools was observed between the three farms (A–C).

Among the pool fecal samples from sows, collected from farm A (n = 12) and B (n = 16), those from farm B were positive (4/16, 25%; 95% CI: 7.3–52.4) while those from farm A were negative (Table 1). However, the observed difference between the two farms was not significant (chi-square 3.5; *p* = 0.113).

Due to a limited budget, we performed sequencing of the partial *VP1* on only 19 samples (no. 6 from farm A and C; no. 7 from farm B) out of the 67 positive for PSV by RT-PCR of the 5′ UTR. 

**Figure 1:**
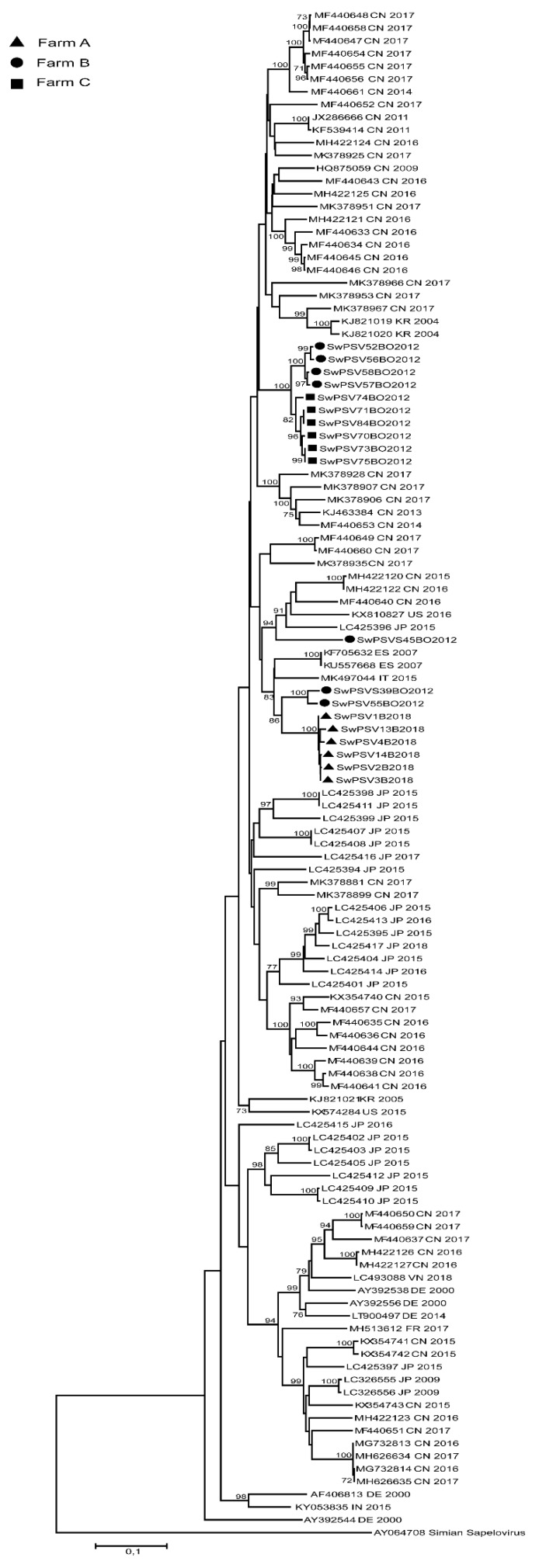
Maximum likelihood phylogenetic tree built with GTR + G + I substitution model, by 1000 resampling, using 100 PSV partial *VP1* sequences (790 nt) including the 19 PSV Italian strains reported in this study. Simian Sapelovirus strain was used as outgroup and bootstrap replicates >70% were reported. Each entry includes accession number and the country origin of strains.

Overall, the phylogenetic tree based on the *VP1* gene showed that the Italian sequences formed three clusters. The sequences within each cluster shared an nt. id. >91% (Figure 1). No nucleotide identity higher than 88% was observed among the Italian strains detected in this study and PSV sequences available in the public database, including the only full genome sequence previously detected in Italy (DIAPD5469-10, MK497044).

On farm A and C, closely related sequences were retrieved, within each farm, the six sequences from farm A and the six from farm C were almost identical (>98.5% nt. id.) (Figure 1). Diversely, the seven sequences from farm B resulted in the three separated clusters sharing a low correlation among each other (Figure 1).

One cluster was formed by two *VP1* sequences from farm B named SwPSV55BO2012 and SwPSVS39BO2012 that shared 97% nt. id. to each other. The second cluster was formed by four sequences (SwPSV52BO2012, SwPSV56BO2012, SwPSV57BO2012, SwPSV58BO2012) sharing 99.3% nt. id. while one sequence (SwPSVS45BO2012) detected in the fecal pool from a sow was in a separate lineage from the other six sequences from the same farm sharing only 85% nt. id. from the other farm B sequences (Figure 1).

It is noteworthy that on farm B sequences highly correlated, sharing 97% nt. id. (SwPSVS39BO2012 and SwPSV55BO2012) were detected in both sows and young growers (Figure 1).

**Figure 2:**
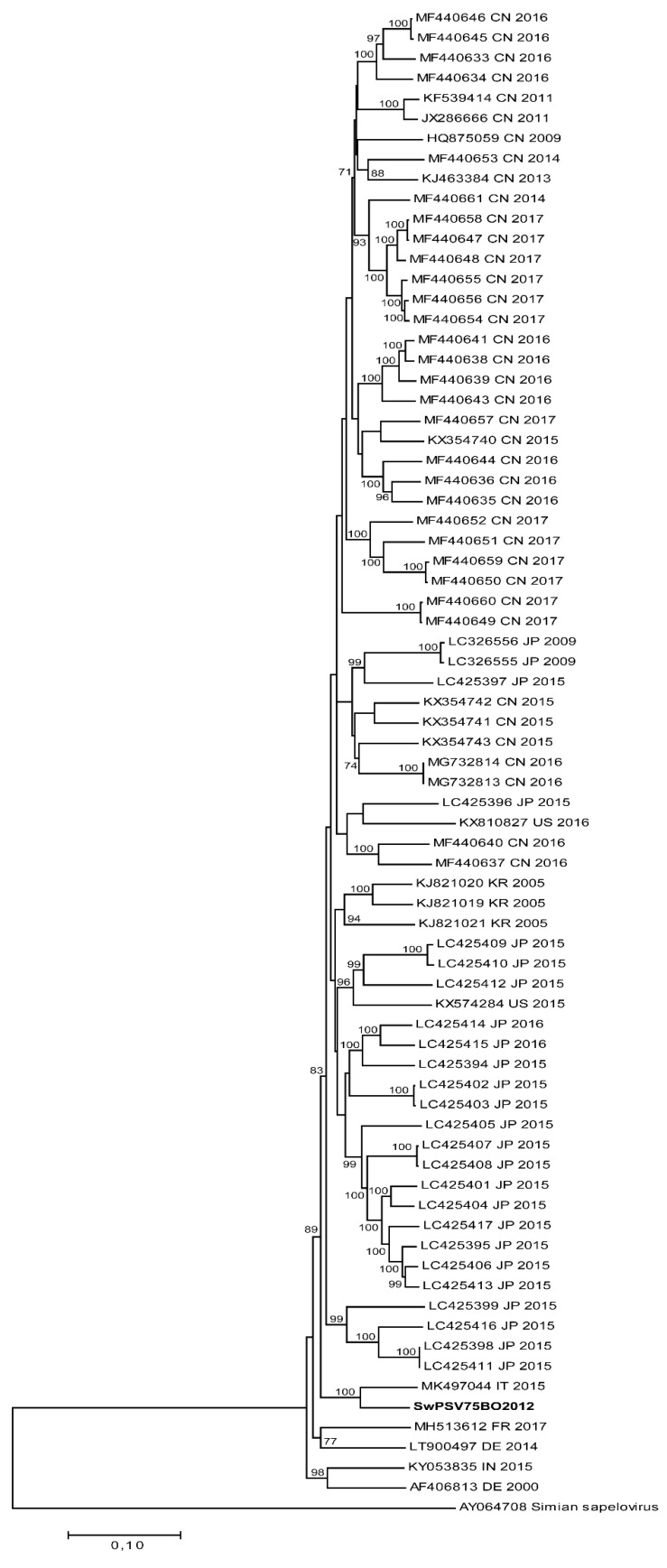
Maximum likelihood phylogenetic tree built with GTR+G+I substitution model, by 1000 resampling, using 73 PSV complete genomes and the Italian complete genome SwPSV75BO2012 sequenced in this study, indicated in bold. Simian Sapelovirus strain was used as outgroup and bootstrap replicates >70% were reported. Each entry includes accession number, the country origin of strains, and collection date.

Two out of the three fecal samples sequenced by the metagenomic approach produced few PSV reads making the complete genome reconstruction impossible. One NGS run, from the sample named SwPSV75BO2012, belonging to an animal housed on farm C produced 2,561,799 reads of which 0.02% (849 reads) were classified as PSV which enabled the attainment of the complete genome with a mean coverage rate of 30.6×. The SwPSV75BO2012 full genome is 7560 nt length and shows the typical PSV genome organization with a 5′ UTR of 455 nt, an ORF of 7014 nt, and the 3′ UTR of 91 nt. The phylogenetic analysis of the complete genome showed a distinctive clustering of SwPSV75BO2012 with the Italian strain DIAPD5469-10, the sole full genome of PSV from Italy available online, sharing 91.7% nt. id. (98% aa. id. on complete ORF) and <86% nt. id. with all the other PSV genomes reported online (NCBI database) (Figure 2). The same tree topology and the correlation between SwPSV75BO2012 and DIAPD5469-10 has also been observed using the 3CD genomic region.

## 4. Discussion

In this study, the occurrence of PSV was evaluated on three farms in Northern Italy, testing pooled fecal samples from two age-category groups of pigs (young growers or sows) by RT-PCR. The occurrence of PSV was frequent, the virus was detected on the three farms with 97–100% positive pools in young growers. Besides the limited number of farms investigated, the presence of PSV was apparently higher than reported in previous studies in both India and Spain, [9,14] but similar to the results reported in Hungary [9]. These data confirm a wide circulation of PSV worldwide but with some geographical differences that may be linked to the epidemiology of infections or farm management procedures.

Our results indicate that PSV is largely widespread especially among younger animals confirming previous findings observed in Korea [15] Spain [14], the Czech Republic [11], and Hungary [9]. In general, circulation in older animals is not detected or occurs less frequently (Spain and the Czech Republic) [11,14]. Sows could be negative for PSV as they are protected by the immune response, which may develop after the exposure to the virus which occurs at a younger age. Nevertheless, a recent study supports the hypothesis that sows are a source of PSV infection in piglets [9].

Among the PSV positive sows detected on farm B (4/16), two different sequences were retrieved. One was only detected in sows (SwPSVS45BO2012) while the other (97% nt.id.) was detected in both young animals (SwPSV55BO2012) and a sow (SwPSVS39BO2012). Young animals and sows are housed in two different buildings. The flow of viral strain, as revealed by sequencing, could have occurred through a lack of adequate hygienic measures, which has caused cross-contamination between the two buildings possibly carried by workers. Another hypothesis is that the infection from sows to piglets that has persisted up to weaner age may be caused by vertical transmission.

The high heterogeneity of PSV sequences on the same farm (farm C) may also be linked to the co-infection of different PSV strains in the same animal [14,16]. However, we cannot rule out that it occurred since no individual feces has been analyzed.

PSV has mainly been detected in the feces of clinically healthy animals in many countries, but it is important to remark that the presence of asymptomatic infections is a possible risk of the virus spreading among animals and that PSV could also be an agent of serious diseases such as polioencephalomyelitis [5].

Phylogenetic analysis was conducted using the complete *VP1* gene sequences, obtained by young growers from each farm and two pools of fecal samples from the sows of farm B, and revealed that Italian strains had high genetic diversity being distant from the other PSV strain sequences available in the GenBank database. This can be explained by the fact that the *VP1* is a hypervariable region because it contains the major neutralization sites of the picornavirus capsid proteins [24].

Due to the absence of classification criteria, PSVs could not be classified into genotypes at present [25]. Nevertheless, in our study, the phylogenetic analysis showed three independent clusters of Italian *VP1* sequences not related to the farm of origin. Two clusters were only formed by Italian strains, distant from all other sequences. This result could be linked to the limited number of sequences available, to the high genetic heterogeneity of PSVs and to the large movement of pigs across European countries, which determines no geographical correlation of the detected strains.

One PSV complete genome was obtained with NGS using the metagenomics approach during a study on HEV surveillance [21]. Despite NGS analyses of two other samples from farm C, only short fragments of the PSV genome were obtained, which may be due to the low titer of PSV in the analyzed pooled fecal samples.

Based on the *VP1* sequences, the Italian PSV strain (SwPSV75BO2012) for which the full genome was obtained, was distant from the Italian strain DIAPD5469-10, the sole other genome available, obtained previously from a pig in Northern Italy [20]. Whereas, in the tree built using the 3CD region (data not showed) from the two strains, they were closely related confirming previous findings that the capsid protein (VP1) and the 3CD regions evolved independently [2,25]. This result should be carefully considered to establish the criteria of classification of PSV which could be different depending on the analyzed genome regions.

## 5. Conclusions

The results obtained in this study demonstrate that PSV may circulate frequently in Italy: only three farms were investigated and all were positive. Several different PSV sequences were detected on the same or different farms. These findings provide new evidence on PSV circulation in Italian farms and new insight on mechanisms of the evolution of PSVs.

## Figures and Tables

**Table 1:** Sapelovirus (PSV) RNA detection in pooled fecal samples from the three examined farms.

Farm	Category	PSV RNA in Pooled Fecal Samples (%)		Total
		Positive	Negative	
A	Young growers	15 (100.0)	0 (0.0)	15
	Sows	0 (0.0)	12 (100.0)	12
	Total	15 (55.6)	12 (44.4)	27
B	Young growers	16 (100.0)	0 (0.0)	16
	Sows	4 (25.0)	12 (75.0)	16
	Total	20 (62.5)	12 (37.5)	32
C	Young growers	32 (97.0)	1 (3.0)	33
	Total	32 (97.0)	1 (3.0)	33
Total	Young growers	63 (98.4)	1 (1.6)	64
	Sows	4 (14.3)	24 (85.7)	28
	Total	67 (72.8)	25 (27.2)	92

## Data Availability

The data generated and/or analyzed during the current study are available in the NCBI database repository, https://www.ncbi.nlm.nih.gov/pubmed/.
